# Ticks infected via co-feeding transmission can transmit Lyme borreliosis to vertebrate hosts

**DOI:** 10.1038/s41598-017-05231-1

**Published:** 2017-07-10

**Authors:** Alessandro Belli, Anouk Sarr, Olivier Rais, Ryan O. M. Rego, Maarten J. Voordouw

**Affiliations:** 1 Laboratory of Ecology and Evolution of Parasites, Institute of Biology, University of Neuchâtel, Neuchâtel, Switzerland; 2 Laboratory of Ecology and Epidemiology of Parasites, Institute of Biology, University of Neuchâtel, Neuchâtel, Switzerland; 3 Institute of Parasitology, ASCR, Biology Centre, Ceske Budejovice, Czech Republic

## Abstract

Vector-borne pathogens establish systemic infections in host tissues to maximize transmission to arthropod vectors. Co-feeding transmission occurs when the pathogen is transferred between infected and naive vectors that feed in close spatiotemporal proximity on a host that has not yet developed a systemic infection. *Borrelia afzelii* is a tick-borne spirochete bacterium that causes Lyme borreliosis (LB) and is capable of co-feeding transmission. Whether ticks that acquire LB pathogens via co-feeding are actually infectious to vertebrate hosts has never been tested. We created nymphs that had been experimentally infected as larvae with *B. afzelii* via co-feeding or systemic transmission, and compared their performance over one complete LB life cycle. Co-feeding nymphs had a spirochete load that was 26 times lower than systemic nymphs but both nymphs were highly infectious to mice (i.e., probability of nymph-to-host transmission of *B. afzelii* was ~100%). The mode of transmission had no effect on the other infection phenotypes of the LB life cycle. Ticks that acquire *B. afzelii* via co-feeding transmission are highly infectious to rodents, and the resulting rodent infection is highly infectious to larval ticks. This is the first study to show that *B. afzelii* can use co-feeding transmission to complete its life cycle.

## Introduction

In vector-borne infections, there are two major pathways by which naive vectors acquire vector-borne pathogens: systemic transmission and co-feeding transmission^1–3^. In systemic transmission, the pathogen establishes a disseminated (systemic) infection in the vertebrate host, which can be acquired by vectors feeding on any part of the body. In co-feeding transmission or non-s﻿ystemic transmission, the pathogen is transmitted between vectors that feed in close proximity to each other on the same host and at the same time^1–3^. In systemic transmission, there is a lag time between when the vertebrate host is first infected and when it can transmit the infection to naive vectors. In co-feeding transmission, vector-to-vector transmission on the vertebrate host is essentially instantaneous. Co-feeding transmission has been reported for a variety of vector-borne pathogens including: Thogoto virus^4^, tick-borne encephalitis virus (TBEV)^2, 3^, the vesicular stomatitis virus^5^, the West Nile virus^6^, *Ehrlichia* bacteria^7, 8^, and *Borrelia burgdorferi* sensu lato (s. l.), the spirochete bacteria that cause Lyme disease^1^.

*Borrelia burgdorferi* s. l. is a genospecies complex of spirochete bacteria that contains the etiological agents of human Lyme disease^9–13^. These tick-borne pathogens are transmitted among vertebrate hosts by hard ticks belonging to the genus *Ixodes*. Numerous studies have shown that the larval stages of *Ixodes* ticks can acquire *B. burgdorferi* s. l. pathogens via co-feeding transmission^14–23^. The importance of co-feeding transmission for Lyme disease pathogens is controversial^1, 18, 24^. A number of theoretical studies have shown that co-feeding transmission makes a modest contribution to the reproduction number (R_0_) of *B. burdorferi* s. l. pathogens^25–27^. A recent study on *B. burgdorferi* sensu stricto (s. s.) suggested that co-feeding transmission facilitated strain co-existence^22^. Recent work on the rodent-adapted *B. afzelii* has shown that the efficacy of co-feeding transmission can vary substantially among strains^19, 28–30^. Strains with a high efficiency of co-feeding transmission appear to have a higher R_0_ value^19^ suggesting that this phenotype is associated with virulence or invasiveness.

To date, no one has tested whether ticks infected via co-feeding transmission are actually infectious to vertebrate reservoir hosts^1^. Live spirochetes have been cultured from co-feeding larval ticks suggesting that the pathogen is viable but not proving that it is actually infectious^16, 23^. Recently, we compared the spirochete load of *B. afzelii* between *I. ricinus* nymphs that acquired this pathogen as larval ticks via co-feeding transmission or systemic transmission^29, 30^. This study found that the spirochete load of *B. afzelii* in co-feeding nymphs was six-fold lower than in systemic nymphs^29, 30^. The size of the spirochete population in the nymphal tick influences the probability that a particular *Borrelia* strain is transmitted to the rodent host^31, 32^. Taken together, these studies suggest that co-feeding nymphs may be less infectious to vertebrate reservoir hosts than systemic nymphs. A recent review pointed out that the critical remaining question is whether nymphs that acquired Lyme disease pathogens as larval ticks via co-feeding transmission are actually infectious to naive vertebrate hosts^1^. In the present study, we compared the infectiousness (i.e. the probability of nymph-to-mouse transmission of *B. afzelii*) between co-feeding nymphs and systemic nymphs (i.e. nymphs that had acquired *B. afzelii* during the larval stage via either co-feeding or systemic transmission). We also tested whether the mode of transmission by which the nymphs had acquired the infection influenced the infection phenotype in the mice and in the next generation of ticks.

## Results

### Efficacy of co-feeding versus systemic transmission

Mice were infested with *B. afzelii*-infected nymphs (5 nymphs per mouse). At 2 and 30 days post nymphal infestation (PNI), the mice were infested with *I. ricinus* larval ticks, and the resulting engorged larval ticks were allowed to moult into nymphs. The larval ticks at 2 days PNI overlapped with the nymphs and acquired *B. afzelii* via co-feeding transmission, whereas the larval ticks at 30 days PNI acquired *B. afzelii* via systemic transmission. For simplicity, these two types of nymphs will hereafter be referred to as the co-feeding nymphs and the systemic nymphs.

**Figure 1:**
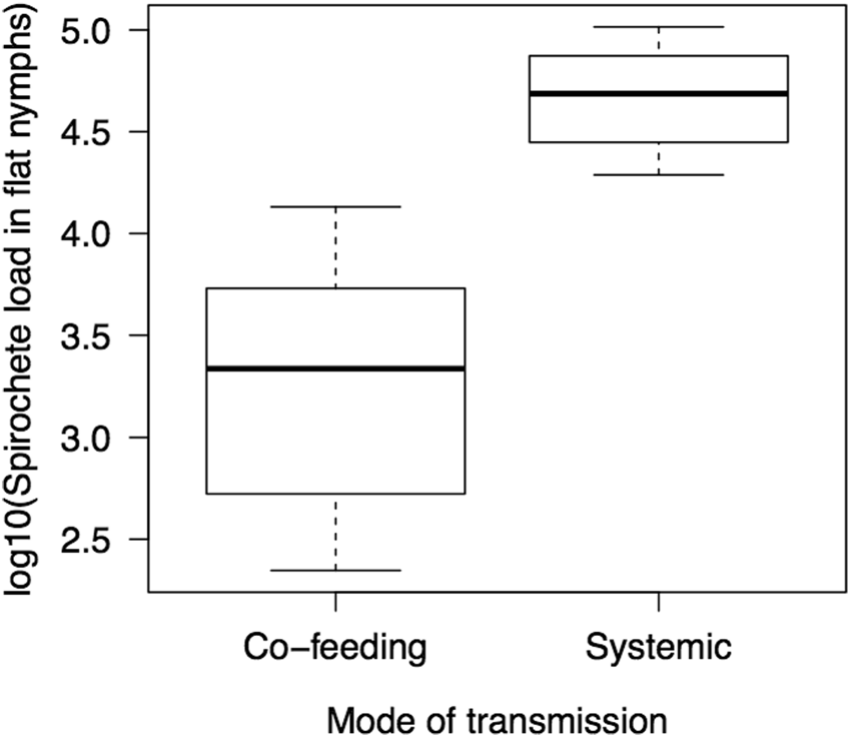
The mode of transmission influenced the spirochete load of *Borrelia afzelii* in the *Ixodes ricinus* nymphs. Nymphs that acquired *B. afzelii* as larvae via systemic transmission had a mean spirochete load that was 26 times higher than nymphs that acquired the infection as larvae via co-feeding transmission. Shown are the medians (black line), the 25th and 75th percentiles (edges of the box), and the minimum and maximum values (whiskers).

A random sample of co-feeding nymphs and systemic nymphs was tested to determine the prevalence of *B. afzelii* in each group of nymphs using qPCR (Table S1 in the supplementary information). The prevalence of *B. afzelii* was 3 times higher for the systemic nymphs (93.3% = 28 infected/30 total) than for the co-feeding nymphs (31.7% = 13 infected/41 total) and this difference was significant (χ^2^ = 24.498, df = 1, p < 0.001). The mean spirochete load in the systemic nymphs (mean = 46379; 95% CI = 24311 to 88477 spirochetes/nymph) was 26 times higher than in the co-feeding nymphs (Fig. 1; mean = 1761; 95% CI = 413 to 7498 spirochetes/nymph) and this difference was significant (Linear mixed effects (LME) model: Δdf = 1, Δdev = 14.626, p < 0.001).

### Infectious challenge of mice with co-feeding nymphs and systemic nymphs

The next step was to test whether the co-feeding nymphs and systemic nymphs differed in their ability to transmit *B. afzelii* to naive mice. Field studies have shown that rodents rarely encounter more than one *B. afzelii*-infected nymph at a time^33–35^. As we wanted our tick-borne infectious challenge to resemble the natural situation, we tried to infest each mouse with an average of 1 *B. afzelii*-infected nymph. For the co-feeding nymphs, the mean prevalence of *B. afzelii* infection was 31.7% (range = 14.3–50.0%) and so each mouse in the co-feeding group was infested with 2 to 9 co-feeding nymphs (Table S2 in the supplementary information). For the systemic nymphs, the mean prevalence of *B. afzelii* infection was 93.3% (range = 80.0–100.0%) and so each mouse in the systemic group was infested with 1 systemic nymph (Table S2 in the supplementary information). Table S2 in the supplementary information shows that the infestations were done in a way so that, on average, each mouse was infested with 1 *B. afzelii*-infected nymph.

### Recovery of the engorged nymphs after nymphal challenge

The co-feeding nymphs and the systemic nymphs were allowed to feed to repletion on the mice. The engorged nymphs were collected and tested for *B. afzelii* infection using qPCR. In any tick infestation experiment, some ticks inevitably go missing because they do not feed on the mouse or because engorged ticks are killed by the mouse. In the co-feeding group, the 26 mice were infested with 90 co-feeding nymphs (mean = 3.5; range = 2–9 nymphs/mouse) and 80 engorged nymphs were recovered (mean = 3.1; range = 1–9 engorged nymphs/mouse). In the systemic group, the 12 mice were infested with 14 systemic nymphs (mean = 1.2; range = 1–2 nymphs/mouse) and 12 engorged nymphs were recovered (mean = 1.0; range = 0–2 engorged nymphs/mouse). In the control group, the 3 mice were infested with 15 uninfected nymphs (mean = 5; range = 5–5 nymphs/mouse) and 13 engorged nymphs were recovered (mean = 4.3; range = 3–5 engorged nymphs/mouse).

Of the 80 engorged nymphs recovered from the mice in the co-feeding group, 17 were infected with *B. afzelii*. Of the 12 engorged nymphs that were recovered from the mice in the systemic group, 7 were infected with *B. afzelii*. In the control group, as expected, none of the 13 engorged nymphs tested positive for *B. afzelii*. In the co-feeding group, we recovered an average of 0.65 engorged *B. afzelii*-infected nymphs per mouse (17 infected nymphs/26 mice = 0.65; range = 0–2 infected nymphs/mouse). In the systemic group, we recovered an average of 0.64 engorged *B. afzelii*-infected nymphs per mouse (7 infected nymphs/12 mice = 0.64; range = 0–1 infected nymphs/mouse). The number of engorged *B. afzelii*-infected nymphs per mouse was compared between the co-feeding and the systemic group and the difference was not significant (Generalized linear model (GLM) with Poisson errors: Δdf = 2, Δdev = 3.746, p = 0.154). Importantly, this result shows that the magnitude of the infectious challenge to which the mice were exposed was the same between the co-feeding group and the systemic group.

### Comparison of the infectious challenge between the co-feeding and systemic treatment

A strict definition for a true infectious challenge is that at least one engorged *B. afzelii*-infected nymph must be recovered from the mouse. In the co-feeding group, at least one engorged *B. afzelii-*infected nymph was recovered for 13 of the 26 mice (50.0%; Table 1). In the systemic group, at least one engorged *B. afzelii-*infected nymph was recovered for 7 of the 12 mice (58.3%; Table 2). Thus, under the strict definition of a true infectious challenge, a similar proportion (50.0% versus 58.3%) of mice was exposed to at least one *B. afzelii-*infected nymph. It is of course regrettable that we failed to recover engorged *B. afzelii*-infected nymphs for a large number of mice (13 + 5 = 18 mice; top﻿ right cells in Tables 1 and 2). However, this outcome is inevitable when tick infestations are done to simulate the natural situation where rodents are exposed to a single infected nymph at a time.

**Table 1:** Contingency table shows the results of challenging mice with the co-feeding nymphs.

*B. afzelii* infection status of the engorged co-feeding nymphs	Mouse remained uninfected after challenge with co-feeding nymphs	Mouse became infected after challenge with co-feeding nymphs	Total
No engorged *B. afzelii*-infected nymphs were recovered from the mouse	8	5^b^	13
At least 1 engorged *B. afzelii*-infected nymph was recovered from the mouse	1	12	13^b^
Total	9	17^a,c^	26^a^

The co-feeding nymphs had been infected as larvae via co-feeding transmission. The rows indicate whether zero or at least one engorged *B. afzelii*-infected co-feeding nymph was recovered following the infectious challenge of the mouse. The columns indicate whether the mouse remained uninfected or developed a systemic *B. afzelii* infection following the infectious challenge with the co-feeding nymphs.

^a^Of the 26 mice in the co-feeding group, 17/26 = 65.4% became infected following the infectious challenge.

^b^Of the 26 mice in the co-feeding group, at least 18 were truly challenged with *B. afzelii*. Proof that a mouse was challenged includes the recovery of at least 1 engorged *B. afzelii*-infected nymph (n = 13) and/or the mouse develops a systemic infection after the challenge (n = 5).

^c^Thus of the 18 mice in the co-feeding group that were truly challenged with *B. afzelii*, 17/18 = 94.4% developed a systemic infection with *B. afzelii*.

**Table 2:** Contingency table shows the results of challenging mice with the systemic nymphs.

*B. afzelii* infection status of the engorged systemic nymphs	Mouse remained uninfected after challenge with systemic nymphs	Mouse became infected after challenge with systemic nymphs	Total
No engorged *B. afzelii*-infected nymphs were recovered from the mouse	2	3^b^	5
At least 1 engorged *B. afzelii*-infected nymph was recovered from the mouse	0	7	7^b^
Total	2	10^a^	12^a^

The systemic nymphs had been infected as larvae via systemic transmission. The rows indicate whether zero or at least one engorged *B. afzelii*-infected systemic nymph was recovered following the infectious challenge of the mouse. The columns indicate whether the mouse remained uninfected or developed a systemic *B. afzelii* infection following the infectious challenge with the systemic nymphs.

^a^Of the 12 mice in the systemic group, 10/12 = 83.3% became infected following the infectious challenge.

^b^Of the 12 mice in the systemic group, at least 10 were truly challenged with *B. afzelii*. Proof that a mouse was challenged includes the recovery of at least 1 engorged *B. afzelii*-infected nymph (n = 7) and/or the mouse develops a systemic infection after the challenge (n = 3).

^c^Thus of the 10 mice in the systemic group that were truly challenged with *B. afzelii*, 10/10 = 100.0% developed a systemic infection with *B. afzelii*.

### Infection status of mice infested with co-feeding and systemic nymphs

Following infestation with the co-feeding nymphs and the systemic nymphs, the infection status of the mice was tested using five different criteria. Blood samples and ear tissue biopsies were taken at 39 days post nymphal infestation (PNI), and all the mice were sacrificed at 59 days PNI and dissected for their heart, bladder, and ventral skin. The blood samples were tested for the presence of *Borrelia*-specific IgG antibodies using a commercial Lyme borreliosis ELISA. The tissue samples of the ear, heart, bladder, and ventral skin were tested for the presence of *B. afzelii* spirochetes using qPCR. A mouse was considered to have developed a systemic infection with *B. afzelii* if it tested positive for one or more of the five criteria: (1) *Borrelia*-specific IgG antibodies, (2) spirochetes in ear, (3) spirochetes in heart, (4) spirochetes in bladder, and (5) spirochetes in ventral skin. Of the 38 mice that were challenged with either co-feeding nymphs or systemic nymphs, 27 tested positive for 3 or more criteria (see Table S3 in the supplementary information). These 27 mice were therefore considered as being systemically infected with *B. afzelii*. The remaining 11 mice tested negative for all five criteria and were therefore considered as not systemically infected with *B. afzelii*. As expected, the 3 mice in the control treatment tested negative for all five criteria (Table S3).

### Comparison of tick-to-mouse transmission of *B. afzelii* between co-feeding nymphs and systemic nymphs

We had previously shown using the number of engorged *B. afzelii*-infected nymphs that the magnitude of the infectious challenge to which the mice were exposed was the same between the co-feeding group and the systemic group. When all the mice in the study were considered, the percentage of mice that became systemically infected with *B. afzelii* was similar between the co-feeding group (65.4% = 17 infected/26 total; Table 1) and the systemic group (83.3% = 10 infected/12 total; Table 2). This difference in nymph-to-mouse transmission of *B. afzelii* between the two types of nymphs was not significant (Proportion test: χ^2^ = 0.056, df = 1, p = 0.454). A more conservative estimate of the probability of nymph-to-mouse spirochete transmission would include only those mice for which there was definitive proof that they had been exposed to *B. afzelii*. The criteria of definitive proof include recovery of at least one engorged *B. afzelii*-infected nymph and/or evidence that the mouse is systemically infected with *B. afzelii*. When the analysis was restricted to the subset of mice for which there was definitive proof that they were exposed to *B. afzelii*, the percentage of systemically infected mice was similar between the co-feeding treatment (94.4% = 17/18; Table 1) and the systemic treatment (100.0% = 10/10; Table 2) and this difference in nymph-to-mouse transmission was not significant (Proportion test: χ^2^ = 0.000, df = 1, p = 1.000). In summary, our study found that the probability of nymph-to-mouse transmission of *B. afzelii* was the same between co-feeding nymphs and systemic nymphs. In other words, co-feeding nymphs and systemic nymphs were equally infectious to naive mice.

### Comparison of the *Borrelia* infection phenotype between mice in the co-feeding versus the systemic treatment

We were interested to test whether the spirochetes transmitted by the co-feeding nymphs would establish a phenotypically different systemic infection in the mice compared to the spirochetes transmitted by the systemic nymphs. In addition to the five criteria that were used to determine whether the mice were systemically infected, we also quantified the probability of mouse-to-nymph (systemic) transmission. In brief, at 42 days PNI, all mice were infested with 50 xenodiagnostic larval ticks and the engorged larval ticks were allowed to moult into xenodiagnostic nymphs. Six weeks after the larva-to-nymph moult, we randomly selected 10 xenodiagnostic nymphs from each mouse and tested them for infection with *B. afzelii* using qPCR. We therefore compared the following 6 infection phenotypes between the mice in the co-feeding group and the systemic group: (1) the *Borrelia*-specific IgG antibody response, the spirochete abundance in the (2) ear, (3) heart, (4) bladder, and (5) ventral skin, and (6) the probability of systemic transmission of *B. afzelii* to xenodiagnostic nymphs.

**Figure 2:**
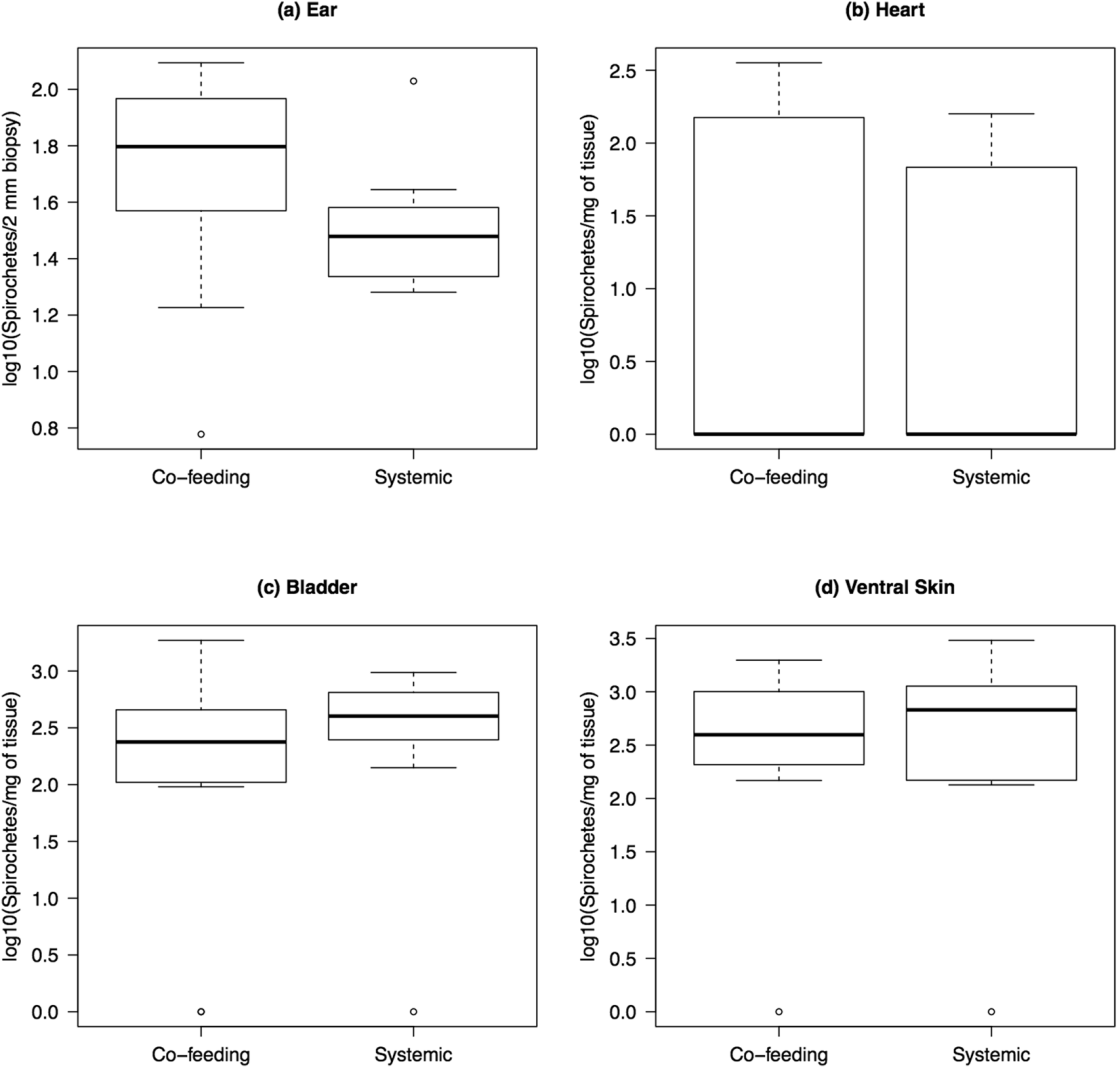
The spirochete load of *B. afzelii* per mg of mouse tissue (log10-transformed) is compared between mice that were infected with co-feeding nymphs versus systemic nymphs in four organs: (**a**) ear, (**b**) heart, (**c**) bladder, and (**d**) ventral skin. There was no difference between the two groups of mice in the mean log10-transformed spirochete load for the ear, heart, bladder, and ventral skin. Shown are the medians (black line), the 25th and 75th percentiles (edges of the box), the minimum and maximum values (whiskers), and the outliers (circles).

The *B. afzelii* spirochete loads in the tissues of the mice were similar between the co-feeding treatment and the systemic treatment. In what follows, the spirochete load is measured in units of spirochetes per mg of mouse tissue in all tissues, except for the ear where it is measured as the number of spirochetes per ear biopsy (2 mm diameter). For the ear, the mean spirochete load of the co-feeding treatment (mean = 52; 95% CI = 37–74) was 1.6 times higher than the systemic treatment (mean = 32; 95% CI = 20–50), but this difference was not significant (Fig. 2; t-test: t = 1.749, df = 25, p = 0.093). For the heart, the mean spirochete load of the co-feeding treatment (mean = 10; 95% CI = 3–35) was 2.5 times higher than the systemic treatment (mean = 4; 95% CI = 1–21), but this difference was not significant (Fig. 2; t = 0.909, df = 25, p = 0.372). For the ventral skin, the mean spirochete load of the co-feeding treatment (mean = 325; 95% CI = 125–844) was very similar to the systemic treatment (mean = 330; 95% CI = 95–1146), and this difference was not significant (Fig. 2; t = −0.020, df = 25, p = 0.984). For the bladder, the mean spirochete load of the co-feeding treatment (mean = 155; 95% CI = 56–432) was 1.5 times lower than the systemic treatment (mean = 233; 95% CI = 62–886), but this difference was not significant (Fig. 2; t = −0.499, df = 25, p = 0.622). The total number of spirochetes in the mice was estimated to be 954,468 spirochetes per mouse (95% CI: 812,310 to 1,121,506 spirochetes per mouse; see the supplementary information). A recent study estimated that *I. ricinus* nymphs inoculate ~100 spirochetes into the rodent host during the nymphal blood meal^36^. These estimates suggest that the *B. afzelii* population increased 10,000-fold in the mice after inoculation by the nymphal tick.

**Figure 3:**
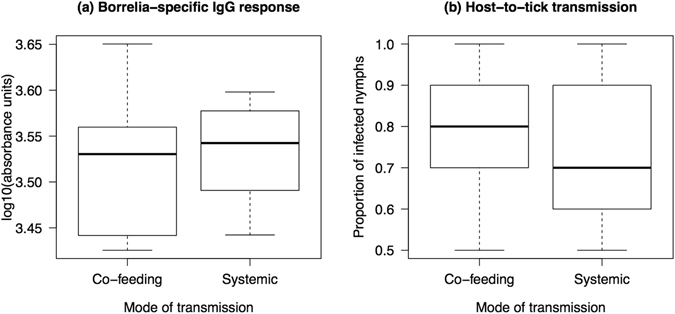
Co-feeding nymphs and systemic nymphs induce the same *B. afzelii* infection phenotype in mice. (**a**) The two types of nymph (co-feeding versus systemic) induced a similar *Borrelia*-specific IgG antibody response in the mice. (**b**) Mice infected with co-feeding nymphs or systemic nymphs had similar levels of host-to-tick (systemic) transmission to xenodiagnostic ticks. Shown are the medians (black line), the 25th and 75th percentiles (edges of the box), and the minimum and maximum values (whiskers).

The strength of the *Borrelia*-specific IgG antibody response was similar between the mice infected with co-feeding nymphs (3283; range = 2664–4471 absorbance units) and the mice infected with systemic nymphs (3404; range = 2768–3964 absorbance units), and the difference was not significant (Fig. 3; t-test: t = 0.608, df = 25, p = 0.549). Host-to-tick (systemic) transmission from the infected mice to the xenodiagnostic ticks was similar between the co-feeding treatment (77.0%; range = 50.0–100.0%) and the systemic treatment (70.0%; range = 50.0–100.0%) and the difference was not significant (Fig. 3; GLM with binomial errors: Δdf = 1, Δdev = 0.882, p = 0.348). The mean spirochete load of the xenodiagnostic nymphs that had fed as larvae on the mice in the co-feeding group was 1.5 times higher (mean = 2125; 95 CI = 1519–2971 spirochetes per nymph) than that of the xenodiagnostic nymphs that had fed as larvae on the mice in the systemic group (mean = 1412; 95 CI = 683–2922 spirochetes per nymph), but this difference was not significant (t-test: t = 1.299, df = 24, p = 0.206). We had previously shown that during the larval blood meal, larval ticks acquire about 100 *B. afzelii* spirochetes from an infected mouse^37^. Thus the spirochete population in the xenodiagnostic ticks increased 14-fold to 21-fold during the transition from engorged larva to flat nymph.

Eight to 10 months after the larva-to-nymph moult, some of the xenodiagnostic nymphs that were still alive were placed into BSK culture to test if the *B. afzelii* spirochete population was still viable. We recovered live spirochetes from 23 xenodiagnostic nymphs that had acquired their *B. afzelii* infection (via systemic transmission at 39 days PNI) from 19 different mice. Of these 19 mice, 14 had been infected with co-feeding nymphs and 5 had been infected with systemic nymphs. In summary, co-feeding nymphs were able to establish a systemic infection in mice that was subsequently acquired by xenodiagnostic larval ticks that were allowed to moult and the resultant xenodiagnostic nymphs contained viable spirochete populations 8–10 months after the larva-to-nymph moult.

## Discussion

**Figure 4:**
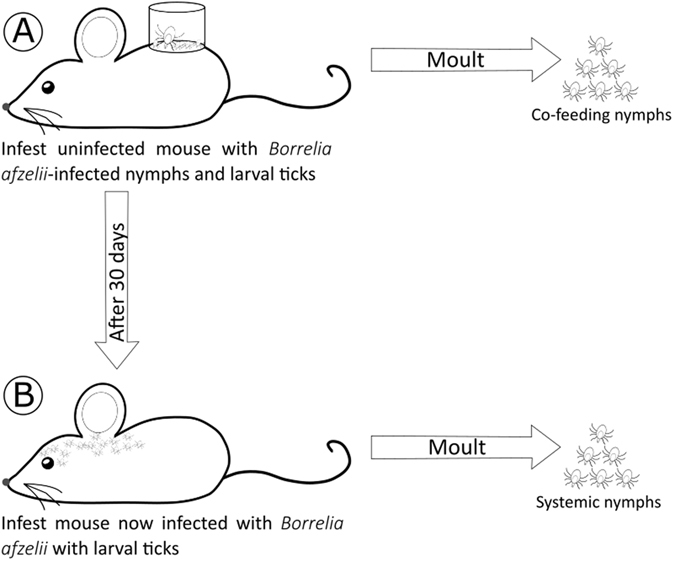
The production of the co-feeding nymphs and the systemic nymphs is shown. (**A**) Each pathogen-free mouse was infested with 5 *B. afzelii*-infected nymphs on day 0 and with 100 larvae on day 2. The larval ticks acquired *B. afzelii* via co-feeding transmission. The engorged larvae were allowed to moult into what are hereafter referred to as the co-feeding nymphs. The nymphs and larvae were placed in a plastic capsule to enhance co-feeding transmission. (**B**) At 30 days after the nymphal infestation, the mice had developed a systemic infection with *B. afzelii*. Each *B. afzelii*-infected mouse was infested with 100 larvae on day 30. The larval ticks acquired *B. afzelii* via systemic transmission. The engorged larvae were allowed to moult into what are hereafter referred to as the systemic nymphs.

**Figure 5:**
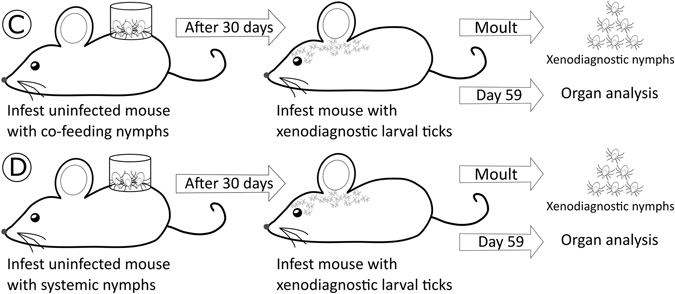
The design of the infection experiment to compare nymph-to-host transmission of *B. afzelii* between the co-feeding nymphs and the systemic nymphs is shown. (**C**) Each of 26 pathogen-free mice was infested with 2 to 9 co-feeding nymphs. (**D**) Each of 12 pathogen-free mice was infested with 1 to 2 systemic nymphs. Nymphs were placed in a capsule to protect them from mouse grooming and to prevent them from escaping. The engorged nymphs were collected and their *B. afzelii* infection status was determined using qPCR. At 30 days after the nymphal infestation, all the mice were infested with 50 xenodiagnostic larvae, and the engorged larval ticks were allowed to moult into xenodiagnostic nymphs. These nymphs were used to quantify host-to-tick transmission. At 59 days after the nymphal infestation, the mice were sacrificed and the organs were dissected to determine the mouse infection status.

The most important result of the present study is our experimental demonstration that *I. ricinus* nymphs that acquire the *B. afzelii* infection as larval ticks via co-feeding transmission (or non-systemic transmission) are infectious to vertebrate reservoir hosts. Two previous studies had shown that live spirochetes could be cultured in BSK-II media from engorged larval ticks one week after they had co-fed with *Borrelia*-infected nymphs^16, 23^ (i.e. in Fig. 4, the engorged larval ticks from mouse A were placed into BSK-II media). While these studies showed that the *Borrelia* pathogens were viable^16, 23^, they did not prove that the spirochetes were actually infectious to naive vertebrate hosts^1^. We extended this past work by allowing the engorged larval ticks to moult into nymphal ticks (i.e. the co-feeding nymphs) and by going through one complete cycle of Lyme borreliosis (i.e. Figs 4 and 5). The co-feeding nymphs established a viable systemic infection in the mice and the xenodiagnostic nymphs as shown by several lines of evidence. First, the spirochetes disseminated from the dorsal skin (where the capsule and nymphs were placed) to multiple organs including the ear, heart, bladder, and ventral skin (Fig. 2). Second, our estimates of the total spirochete load in the mice suggest that the spirochete population expanded 10,000-fold following inoculation into the mice by the co-feeding nymphs and systemic nymphs (see the supplementary information). Third, the spirochetes induced a strong *Borrelia*-specific antibody response (Fig. 3a). Fourth, the subset of infected mice had high mouse-to-tick transmission (~70.0%) to xenodiagnostic ticks at 39 days after the infectious challenge (Fig. 3b). Fifth, the spirochete population of the xenodiagnostic ticks expanded 14-fold to 21-fold during the transition from the engorged larva to the flat nymph. Sixth, live spirochete populations were recovered from these xenodiagnostic ticks 8–10 months later. Taken together, these observations suggest that our co-feeding ticks established a viable, systemic infection in the mice and the xenodiagnostic nymphs. Our study also suggests that regardless of whether the larval ticks acquired the infection via co-feeding transmission or systemic transmission, the resulting nymphs are equally infectious to naive vertebrate hosts. To date, all theoretical work on the epidemiology of LB has assumed that co-feeding nymphs and systemic nymphs are equally infectious to vertebrate hosts^2, 25–27, 38, 39^. The present study confirms that this assumption is justified.

The importance of co-feeding transmission for the fitness of LB pathogens has been debated^1, 2, 18, 24, 40^. Theoretical models have shown that co-feeding transmission increases the reproduction number (R_0_) of LB pathogens by a modest amount (2.07–6.68%) depending on the ecological circumstances, such as the degree of tick aggregation and the proportion of *Borrelia*-competent hosts^25–27^. Thus, while co-feeding transmission is critical for the epidemiology of other tick-borne pathogens such as tick-borne encephalitis virus^2, 3^, this mode of transmission is not necessary for the maintenance of LB pathogens in nature^27^. Randolph *et al*.^2^ suggested that co-feeding allows *Borrelia* pathogens to achieve transmission on incompetent vertebrate hosts, and there is some indirect evidence for this idea in sheep and deer^41, 42^. In contrast, we used experimental infections with *B. afzelii* isolate NE4049 to show that the efficacy of co-feeding transmission was greatly reduced on incompetent hosts such as rodents immunized with a protective *Borrelia* antigen^29^ and two species of passerine bird^43^. These studies suggest that *B. afzelii* cannot use co-feeding as an effective strategy to achieve transmission on *Borrelia*-incompetent hosts.

In nature, *Borrelia* strains capable of co-feeding transmission might have higher fitness than strains that are not capable of this mode of transmission^1, 19, 22, 29, 30^. All else being equal, strains that have co-feeding transmission can start reproducing earlier than strains that rely exclusively on systemic transmission^1, 22, 30, 44^. Effective co-feeding transmission requires rapid expansion of the spirochete population in the host skin so that ticks feeding in close proximity to the infected tick acquire the pathogen^1^. This feature suggests that co-feeding may be associated with other strain-specific phenotypes such as pathogen burden, invasiveness, or pathology. Comparison of the *B. afzelii* isolate used in the present study (NE4049) to a reference isolate (E61) that lacks co-feeding transmission, found that NE4049 establishes a higher spirochete load in both the rodent tissues and the nymphal ticks^20, 29^. We also showed that isolate NE4049 has a higher lifetime transmission success than isolate E61^30^. In contrast, a recent study on *B. burgdorferi* s. s. in the United States found the opposite pattern^22^. In that study, the isolate that was capable of co-feeding transmission (B348) was also rapidly cleared from the mouse^22^. Isolate B348 therefore had lower lifetime transmission success than isolate BBC13, which was not capable of co-feeding transmission but was able to establish a persistent systemic infection in the mouse^22^.

Strains capable of co-feeding transmission might also have a competitive advantage in the context of mixed infections. *Ixodes* ticks in the field are often infected with multiple *Borrelia* strains^32, 45–49^. Thus mice are frequently bitten by nymphs carrying multiple *Borrelia* strains, and there is evidence suggesting that these strains experience competition in the rodent reservoir host^44, 50, 51^. Strains capable of co-feeding transmission would benefit from having early transmission to larval ticks before having to compete with other strains during the later, systemic phase of the infection^1, 22^. We have recently shown using experimental infections that field-derived isolates of *B. afzelii* differ in their efficacy of co-feeding transmission^19, 29, 30^. Interestingly, strains with high co-feeding transmission also had the highest values of the reproduction number (R_0_)^19^. The strain-specific R_0_ values estimated in this laboratory study were subsequently used to explained differences in prevalence among strains of *B. afzelii* in a long-term field study on a wild population of *I. ricinus* nymphs^52^. Isolate NE4049 used in the present study carries *ospC* major group (oMG) A10, and strains that carry this oMG are the most common strains in wild populations of *I. ricinus* ticks and rodent reservoir hosts^19, 28, 32, 48, 52^. In summary, strains capable of co-feeding transmission have higher fitness in a laboratory Lyme disease system and are more common in wild tick populations.

The probability of nymph-to-host transmission of *Borrelia* pathogens increases with the duration of tick attachment^53^. During the nymphal blood meal, the spirochete load increases dramatically in the tick midgut^20, 54–60^ and the spirochetes migrate to the tick salivary glands^59, 61^. This spirochete migration is why the risk of acquiring a *Borrelia* infection increases with the duration of time that the tick remains attached to the host^53, 59, 62–64^. In the present study, the probability of nymph-to-host transmission of *B. afzelii* was very high. For the subset of mice for which there was proof that they had been challenged with at least one *B. afzelii*-infected nymph, the probability of nymph-to-host transmission was 94.4% for the co-feeding nymphs and 100.0% for the systemic nymphs (Tables 1 and 2). The high probability of nymph-to-host transmission of *B. afzelii* in the present study was probably caused by our infestation protocol that allows nymphs to feed to repletion on the mice (period of 4 to 5 days). In future experiments, removing the nymphal ticks after 2 or 3 days of feeding may reveal differences in the probability of nymph-to-host transmission of *B. afzelii* between co-feeding nymphs and systemic nymphs.

Co-feeding transmission (31.7% = 13/41; Table S1) was 3 times lower than systemic transmission (93.3% = 28/30; Table S1). This result was similar to our previous work on *B. afzelii* isolate NE4049, which found a two-fold difference between the two modes of transmission^19, 29, 30^. The spirochete load in the co-feeding nymphs was 26 times lower than that in the systemic nymphs (Fig. 1). This result was similar to our previous work on *B. afzelii* isolate NE4049, which found a six-fold difference in spirochete load between the two modes of transmission^29, 30^. These data suggest that larval ticks acquire fewer spirochetes via co-feeding than systemic transmission and/or that co-feeding spirochetes have a slower growth rate than systemic spirochetes^29^. An alternative explanation is the difference in experimental conditions between the two modes of transmission in the present and the previous study^29^. To facilitate co-feeding transmission, the 100 larvae and the 5 nymphs were placed in a capsule (15 mm diameter) and forced to feed on a limited area of skin. These conditions may have induced a strong local inflammatory response in the mouse skin^65^, which then had a negative effect on the spirochete population inside the co-feeding larval ticks. In contrast, the systemic larval ticks were able to move around and attach anywhere on the body of the mouse. Thus differences in the tick density per unit area of mouse skin and in the intensity of the resulting mouse inflammatory response were probably responsible for the observed 26-fold difference in spirochete load between the co-feeding nymphs and the systemic nymphs. There were no other significant differences in infection phenotype between the two modes of transmission. The spirochete loads in the mouse tissues were similar between the co-feeding and systemic group (Fig. 2). The *Borrelia*-specific IgG antibody response and the mouse-to-tick transmission success to the xenodiagnostic ticks were similar between the co-feeding and systemic group (Fig. 3). In summary, the mode of transmission had a dramatic impact on the spirochete load in the resultant nymphs (Fig. 1), but there were no consequences for the subsequent cycle of LB transmission (Figs 2 and 3).

The transmission success of a vector-borne pathogen often depends on its density in the tissues of the vertebrate host or the arthropod vector. A recent study using genetically tagged strains of *B. burgdorferi* s. s. found that strains with higher spirochete loads in the nymphal tick were more likely to be transmitted to the rodent reservoir host^31^. We had recently shown that strains of *B. afzelii* that have higher spirochete loads in the nymphal tick are also the strains that are the most common in a wild population of *I. ricinus* ticks^32^. This study suggests that the probability of nymph-to-host transmission of *B. afzelii* depends on the nymphal spirochete load^32^. We had therefore expected that the 26-fold lower spirochete load of the co-feeding nymphs would reduce the probability of nymph-to-host transmission compared to the systemic nymphs, but this was not the case. In nature by contrast, the variation in nymphal spirochete load may influence nymph-to-host transmission for a number of reasons. First, many *Borrelia*-infected nymphs may not get to feed to repletion because wild vertebrate hosts often remove ticks via grooming^66^. Under these conditions, strains with higher spirochete loads in the nymphal tick may have higher fitness because they have earlier nymph-to-host transmission. In addition, *Ixodes* ticks in the field are often infected with multiple strains^32, 45–49^. In the context of multiple strain infections, variation in nymphal spirochete load could mediate competition between strains for access to the tick salivary glands and influence strain-specific nymph-to-host transmission success^32, 50^. In summary, nymphal spirochete load could influence nymph-to-host transmission under natural conditions where host grooming interrupts the tick blood meal and/or where *Borrelia* strains compete for access to the tick salivary glands and subsequently to ﻿the vertebrate reservoir host.

The probability of vector-to-host transmission is a critical parameter in the life history of any vector-borne pathogen. To estimate this parameter, it is important to use biologically realistic infectious challenges that mimic the situation in nature. A study on a North American strain of *B. burgdorferi* s. s. found that co-feeding transmission was 5% when mice were infested with unrealistically high densities of immature *I. scapularis* ticks (40 nymphs and 200 larvae)^16^. This result led the authors to conclude that co-feeding transmission was theoretically possible but not ecologically relevant^16^. The purpose of the present study was to test whether co-feeding nymphs could infect naive vertebrate hosts under ecologically relevant conditions. Field studies have shown that wild rodents are rarely infested with more than one *I. ricinus* nymph at a time^33–35^. For this reason, we chose to infest our mice with an average of 1 *B. afzelii*-infected nymph. As the prevalence of *B. afzelii* in the co-feeding nymphs (31.7% = 13/41; Table S1) was much lower than the systemic nymphs (93.3% = 28/30; Table S1), we had to infest the mice with more co-feeding nymphs than systemic nymphs. Analysis of the recovered engorged nymphs found an average of 0.65 and 0.64 engorged *B. afzelii*-infected nymphs per mouse in the co-feeding group and the systemic group, respectively. Thus, we were successful in infesting the mice in the co-feeding group and the systemic group with similar numbers of infected nymphs. In the present study, there were 10 mice (Tables 1 and 2) for which no engorged *B. afzelii*-infected nymphs were recovered and which did not develop a systemic infection. As there was no evidence of exposure to *B. afzelii*, these 10 mice had to be excluded from the estimate of nymph-to-host transmission. Thus the cost of using a realistic nymphal challenge is that a relatively high proportion of mice will not be exposed to any *B. afzelii*-infected nymphs.

## Conclusions

Co-feeding transmission allows *B. afzelii* to complete the Lyme borreliosis life cycle. Nymphs that had acquired *B. afzelii* during the larval blood meal via co-feeding transmission (at 2 days post-infection) were equally infectious to naive rodent reservoir hosts compared to nymphs that had acquired the infection via systemic transmission (at 30 days post-infection). The spirochete load in the co-feeding nymphs was 26 times lower than that of the systemic nymphs. The higher tick density in the co-feeding assay and the stronger inflammatory response in the skin probably caused this reduction in spirochete load in the co-feeding nymphs. This 26-fold difference in spirochete load had no effects on any of the infection phenotypes in the subsequent LB cycle. In summary, our study shows that the mode of transmission by which nymphs had previously acquired the *Borrelia* infection (during the larval blood meal) does not influence their subsequent infectiousness to the vertebrate host.

## Methods

### Mice, Ixodes ricinus ticks, and Borrelia afzelii

Pathogen-free, female *Mus musculus* BALB/c ByJ mice were used as the vertebrate reservoir host. The larval and nymphal ticks came from our laboratory colony of pathogen-free *Ixodes ricinus* ticks. We used *Borrelia afzellii* isolate NE4049, which was obtained from an *I. ricinus* nymph at a field site near Neuchâtel, Switzerland. This strain has multi-locus sequence type ST679, *ospC* major group (oMG) allele A10, and strain ID number 1887 in the *Borrelia* MLST database. We used isolate NE4049 (also referred to as *ospC* strain A10) because it has high co-feeding transmission^19, 29, 30^.

### Ethics statement and animal experimentation permits

Experiments were carried out in accordance with Swiss legislation on animal experimentation. The commission that is part of the ‘Service de la Consommation et des Affaires Vétérinaires (SCAV)’ of Canton Vaud, Switzerland evaluated and approved the ethics of this part of the study. The Veterinary Service of the Canton of Neuchâtel, Switzerland issued the animal experimentation permits (NE2/2016).

### Production of the challenge nymphs

We created *B. afzelii*-infected *I. ricinus* nymphs (hereafter referred to as the challenge nymphs) as follows. Six laboratory mice were infected with *B. afzelii ospC* strain A10 via intraperitoneal or subcutaneous inoculation of ~5,000 spirochetes in 100 μl of PBS. Four weeks post-infection, ~100 pathogen-free larval ticks were fed on each of the six *B. afzelii*-infected mice. The blood-engorged larval ticks were placed in individual Eppendorf tubes, kept at room temperature and 95% relative humidity, and were allowed to moult into nymphs^20, 62^. Six weeks after the moult, these nymphs were used to infect the next group of mice and to create the co-feeding nymphs and the systemic nymphs (see below).

### Production of the co-feeding and systemic nymphs

The production of the co-feeding nymphs and the systemic nymphs is shown in Fig. 4. Each of ten mice was infested with 5 *B. afzelii-*infected challenge nymphs. The nymphs were placed in a plastic capsule (15 mm in diameter) that was attached to the shaved back of each mouse with wax^19, 20^. At 28 days post-nymphal infestation (PNI), *B. afzelii* infection in the mice was confirmed using a *Borrelia*-specific qPCR of ear tissue biopsies and a *Borrelia*-specific ELISA of blood samples. At 2 days PNI, we added ~100 pathogen-free *I. ricinus* larvae to the capsule to co-feed with the challenge nymphs. At 30 days PNI, each mouse was infested with ~100 pathogen-free larvae. All engorged larvae were kept in individual tubes and allowed to moult into nymphs using the protocol described for the challenge nymphs. For simplicity, the two types of nymphs that fed at 2 days PNI and 30 days PNI will hereafter be referred to as ‘co-feeding’ nymphs and ‘systemic’ nymphs, respectively. For each of the 10 mice, we randomly selected 4 co-feeding nymphs and 5 systemic nymphs, froze them at −20 °C at 4 weeks post-moult, and tested them for *B. afzelii* infection using qPCR. In this way, we determined the prevalence of *B. afzelii* infection in the co-feeding nymphs and the systemic nymphs of each mouse.

### Compare tick-to-host transmission between co-feeding nymphs and systemic nymphs

An infection experiment was performed to compare nymph-to-mouse transmission of *B. afzelii* between the co-feeding nymphs and the systemic nymphs (Fig. 5). A total of 41 mice were randomly assigned to one of three infestation treatments: (1) one infected co-feeding nymph (n = 26 mice), (2) one infected systemic nymph (n = 12 mice), and (3) five uninfected nymphs (n = 3 mice). We used a bigger sample size for the co-feeding nymph treatment because we expected nymph-to-mouse transmission to be much lower compared to the systemic nymph treatment. To simulate a natural infectious challenge, each mouse was challenged, on average, with one *B. afzelii*-infected nymph. We used the infection prevalence of the co-feeding nymphs or the systemic nymphs to calculate the number of nymphs required to obtain one infected nymph. For example, if the infection prevalence of the co-feeding nymphs was 20.0% then 5 co-feeding nymphs will contain, on average, one infected nymph. Following the infectious challenge, all recovered engorged nymphs were frozen at −20 °C and analyzed using qPCR do determine their *B. afzelii* infection status.

### Infection phenotype and host-to-tick transmission of mice infected with co-feeding nymphs versus systemic nymphs

At 39 days PNI with the co-feeding, systemic, or uninfected control nymphs, each of the 41 mice was tested for infection using a *Borrelia*-specific qPCR of the ear tissue biopsy and a *Borrelia*-specific ELISA (see below). On day 42 PNI, each mouse was infested with ~100 pathogen-free, xenodiagnostic *I. ricinus* larvae. Engorged larvae were allowed to moult into nymphs, frozen at −20 °C at 4 week post-moult, and tested for infection using a *Borrelia*-specific qPCR (see below). On day 59 PNI, all 41 mice were sacrificed and the heart, bladder, and the ventral skin were dissected under sterile conditions.

### Culture of viable *B. afzelii* spirochetes from xenodiagnostic nymphs

Eight to 10 months after the larva-to-nymph moult, the xenodiagnostic nymphs were checked if they were alive or dead. To test if the *B. afzelii* spirochete populations were still viable, ~4 live nymphs for a subset of 30 mice were placed into BSK culture. After two weeks, the BSK cultures were screened for viable spirochetes using a light microscope. Only BSK cultures that contained motile spirochetes were considered as viable.

### Mouse tissues and blood samples

The ear tissue biopsies were taken from the mice using a 2 mm forceps-type punch as described by ref. 20 and stored at −20 °C. For the heart, bladder, and ventral skin, a piece of tissue estimated to weigh ~5 mg was cut from the organ and weighed to determine its exact mass. This approach allowed us to standardize the spirochete load per mg of tissue for these three organs. The blood samples were taken from the saphenous vein. The serum was separated from the blood cells as described by ref. 20 and stored at −20 °C.

### DNA extraction and qPCR assay of nymphs and mouse tissues

To test whether the ticks and mice were infected with spirochetes, we used a qPCR assay that amplifies a 132 bp fragment of the *flagellin* gene of *B. burgdorferi* s. l. pathogens^67^. Total DNA from individual ticks was extracted using the QIAGEN DNeasy® Blood & Tissue Kit and following the protocol described by ref. 20. Total DNA from individual mouse tissues was extracted using the DNeasy Blood&Tissue kit mini spin column following the protocol of the manufacturer. For the ear tissue biopsy, we used the entire sample (2 mm diameter). To quantify the number of *B. afzelii* spirochetes present in each tick or in each mouse tissue we followed the qPCR protocol described by ref. 20.

### Enzyme-linked immunosorbent assay (ELISA)

We used the SERION® ELISA classic *Borrelia burgdorferi* IgG/IgM immunoassay to detect the presence of IgG antibodies against *B. afzelii*. Mouse sera was diluted 1:100 in blocking solution that was composed of 2% bovine serum albumin (BSA) in phosphate-buffered saline (PBS). The ELISA plate was incubated with the mouse sera for 45 min at room temperature. The plate was washed three times with a solution of 0.1% TWEEN in PBS for 5 min. The plate was incubated with a goat anti-mouse IgG horseradish peroxidase conjugate diluted 1:5000 in the blocking solution for 45 min at room temperature. The plate was washed three times with PBS-TWEEN as described above. The final step was to add 100 μl of tetramethylbenzidine (TMB) to each well. The absorbance at a wavelength of 652 nm was measured every two minutes for one hour to determine the strength of the IgG antibody response.

## Statistical Methods

All statistical analyses were done in R version 3.2.3^68^.

### *B. afzelii* infection status and spirochete load in *I. ricinus* nymphs

A nymph was considered infected with *B. afzelii* if it contained >1 spirochete. The nymphal spirochete load refers to the number of spirochetes in the nymphal tick. As qPCR is an exponential process, all spirochete loads were log10-transformed to improve the normality of the data. For each mouse and mode of transmission (co-feeding vs. systemic), the mean log10-transformed nymphal spirochete load was calculated for the subset of infected ticks (i.e., the uninfected ticks were excluded).

### Spirochete load in mouse tissues

For each of the three organs (heart, bladder, and ventral skin), the spirochete load was divided by the weight of the tissue. The spirochete load of each tissue refers to the number of spirochetes per milligram of tissue on day 59 PNI. For the ear tissue biopsies, the spirochete load refers to the number of spirochetes per biopsy (2 mm diameter) on day 28 PNI.

### Efficacy of co-feeding versus systemic transmission

The proportion of *B. afzelii*-infected ticks and the spirochete load was compared between the flat co-feeding nymphs and the flat systemic nymphs. A generalized linear mixed effects (GLME) model with binomial errors was used to test whether the mode of transmission (co-feeding vs. systemic) influenced the proportion of infected nymphs. For the subset of infected nymphs, a linear mixed effects (LME) model with normal errors was used to test whether the mode of transmission influenced the mean log10-transformed spirochete load. In the GLME and LME models, the mode of transmission was a fixed factor and mouse identity was a random factor.

### Infection status of mice infested with co-feeding and systemic nymphs

Following infestation with either co-feeding nymphs or systemic nymphs, the infection status of each mouse was determined using five criteria: presence of spirochetes in (1) ear tissue biopsy, (2) heart, (3) bladder, (4) ventral skin, and (5) presence of *Borrelia*-specific IgG antibodies. Mice that tested positive for one or more of these five criteria were considered as systemically infected with *B. afzelii*.

### Comparison of tick-to-mouse transmission of *B. afzelii* between co-feeding nymphs and systemic nymphs

To compare the efficacy of tick-to-mouse transmission between co-feeding nymphs and systemic nymphs, the proportion of infected mice was compared between the co-feeding treatment and the systemic treatment. This comparison assumes that the probability of exposure to a *B. afzelii*-infected nymph was the same between the two groups of mice. A more conservative approach is to restrict the comparison to the subset of mice for which there is definitive proof that they were exposed to *B. afzelii*. Proof that a mouse was exposed to *B. afzelii* includes: (1) recovery of one or more engorged *B. afzelii*-infected nymphs and/or (2) mouse develops a systemic infection following the nymphal challenge.

### Comparison of the *Borrelia* infection phenotype between mice in the co-feeding versus the systemic treatment

Independent samples t-tests were used to compare the log10-transformed spirochete load in the tissues between the co-feeding mice and the systemic mice for each of the four organs: ear, heart, bladder, and ventral skin. An independent samples t-test was used to compare the log10-transformed *Borrelia*-specific IgG antibody response between the co-feeding mice and the systemic mice. A generalized linear model (GLM) with binomial errors was used to compare transmission of *B. afzelii* from infected mice to the xenodiagnostic ticks between the co-feeding group and the systemic group. For each infected mouse, we calculated the mean log10-transformed spirochete load for the subset of infected xenodiagnostic nymphs (i.e. uninfected nymphs were excluded). An independent samples t-test was then used to compare the mean log10-transformed spirochete load in the xenodiagnostic nymphs between the co-feeding mice and the systemic mice.

## Electronic supplementary material

Supplementary information
