# Supplementary material for: Ticks infected via co-feeding transmission can transmit Lyme borreliosis to vertebrate hosts
Source: Sci Rep. 2017 Jul 10;7:5006. doi: 10.1038/s41598-017-05231-1 (PMC5503982; doi:10.1038/s41598-017-05231-1)
Supplement: Supplementary file 1 — Supplementary information [file 41598_2017_5231_MOESM1_ESM.pdf]

Supplementary information

Title: Ticks infected via co-feeding transmission can transmit Lyme borreliosis to vertebrate hosts

Authors: Alessandro Belli, Anouk Sarr, Olivier Rais, Ryan O. M. Rego, and Maarten J. Voordouw

Production of the co-feeding nymphs and the systemic nymphs: Six mice (M11, M15, M16, M18, M19, and M20) were experimentally infected with *B. afzelii* via tick bite. At 2 and 30 days post-nymphal infestation (PNI) these mice were infested with ~100 pathogen-free *I. ricinus* larvae from our laboratory colony. The engorged larval ticks were collected and allowed to moult into nymphs. The larval ticks that acquired the *B. afzelii* spirochetes at 2 and 30 days PNI are hereafter referred to as the co-feeding nymphs and the systemic nymphs, respectively.

Development times of co-feeding and systemic nymphs: The co-feeding larvae and the systemic larvae had different larva-to-nymph moulting times. The co-feeding larvae dropped off the mice on November 12, 2015 and 50% had moulted into nymphs by March 23, 2016. The systemic larvae dropped off the mice on December 11, 2015 and 50% had moulted into nymphs by February 26, 2016. Thus, the larva-to-nymph moulting time of the co-feeding larvae (84 days ± 8 days) was 2 times longer than the systemic larvae (49 days ± 9 days).

Comparison of the efficacy of spirochete acquisition and spirochete load between the co-feeding versus the systemic nymphs: For each of the 6 mice, we checked the proportion of infected ticks for 6–10 co-feeding nymphs and for 5 systemic nymphs (Table S1). We also compared the mean spirochete load between the co-feeding nymphs and the systemic nymphs (Table S1).

Table S1. Systemic transmission of *B. afzelii* is more efficient at infecting ticks than co-feeding transmission. For each of the six mice and for each mode of transmission, the number of infected nymphs divided by the total number of nymphs tested (percentage of infected nymphs is given in brackets), and the mean nymphal spirochete load are shown.

Mouse ID	Co-feeding		Systemic	
	Infected nymphs/ Total nymphs (%)	Spirochete load[a]	Infected nymphs/ Total nymphs (%)	Spirochete load[a]
M11	1/6 (16.7%)	1,004	5/5 (100.0%)	28,066
M15	1/6 (16.7%)	13,510	4/5 (80.0%)	51,478
M16	4/8 (50.0%)	2,165	5/5 (100.0%)	19,398
M18	1/4 (25.0%)	6,053	5/5 (100.0%)	45,961
M19	1/7 (14.3%)	222	4/5 (80.0%)	74,627
M20	5/10 (50.0%)	4,779	5/5 (100.0%)	103,526
Total	13/41 (31.7%)		28/30 (93.3%)	

[a] The geometric mean of the subset of infected nymphs.

Infectious challenge of mice with co-feeding and systemic nymphs: The main experiment compared the efficacy of nymph-to-mouse transmission of *B. afzelii* between co-feeding nymphs and systemic nymphs. In the main experiment, 26 mice were challenged with co-feeding nymphs, 12 mice were challenged with systemic nymphs, and 3 control mice were challenged with uninfected nymphs. The results of the nymphal challenge are shown in Table S2. In the first row for example, mouse 21 (M21) was challenged with 8 nymphs (Total1) that had been infected as larvae via co-feeding transmission after feeding on mouse 11 (M11). The expected probability that these nymphs are infected is 16.7% (Prev), so we expect that 8*0.167 = 1.33 nymphs are infected with *B. afzelii* (Infect1). The expected probability of infection was based a random sample of co-feeding nymphs that was tested for M11 (see Table S1). After the infectious challenge, 7 engorged nymphs were recovered (Total2) of which 2 were positive for *B. afzelii* (Infect2). We therefore conclude that M21 was challenged with at least one *B. afzelii*-infected nymph ('Yes' in the 'Challenge' column).

In contrast, mouse 24 (M24) was challenged with 2 nymphs (Total1), which had an expected probability of infection of 50.0% (Prev) so that the challenge is expected to contain 2*0.50 = 1 infected nymph (Infect1). After the infectious challenge, 2 engorged nymphs were recovered (Total2) of which 0 tested positive for *B. afzelii* (Infect2). We therefore conclude that M24 was not challenged with any *B. afzelii*-infected nymphs ('No' in the 'Challenge' column).

Table S2. Infectious challenge results are shown for the mice infested with co-feeding nymphs, systemic nymphs, and uninfected control nymphs.

		Flat nymphs for infestation				Engorged nymphs recovered		
Mouse ID[a]	Treatment[b]	Origin[c]	Total1[d]	Prev[e]	Infect1[f]	Total2[g]	Infect2[h]	Challenge[i]
M21	Co-feeding	M11	8	16.7%	1.33	7	2	Yes
M22	Co-feeding	M15	9	16.7%	1.50	9	1	Yes
M23	Co-feeding	M16	2	50.0%	1	2	1	Yes
M24	Co-feeding	M16	2	50.0%	1	2	0	No
M25	Co-feeding	M16	2	50.0%	1	2	0	No
M26	Co-feeding	M16	2	50.0%	1	1	0	No
M27	Co-feeding	M16	2	50.0%	1	2	0	No
M28	Co-feeding	M16	2	50.0%	1	2	1	Yes
M29	Co-feeding	M18	4	25.0%	1	4	0	No
M30	Co-feeding	M18	4	25.0%	1	4	0	No
M31	Co-feeding	M19	7	14.3%	1	7	0	No
M32	Co-feeding	M19	7	14.3%	1	3	0	No
M33	Co-feeding	M19	7	14.3%	1	7	2	Yes
M34	Co-feeding	M19	8	14.3%	1.14	8	1	Yes
M35	Co-feeding	M20	2	50.0%	1	1	0	No
M36	Co-feeding	M20	2	50.0%	1	2	1	Yes
M37	Co-feeding	M20	2	50.0%	1	2	0	No
M38	Co-feeding	M20	2	50.0%	1	2	2	Yes
M39	Co-feeding	M20	2	50.0%	1	2	0	No
M40	Co-feeding	M20	2	50.0%	1	1	0	No
M41	Co-feeding	M20	2	50.0%	1	2	1	Yes
M42	Co-feeding	M20	2	50.0%	1	1	1	Yes
M43	Co-feeding	M20	2	50.0%	1	2	1	Yes
M44	Co-feeding	M20	2	50.0%	1	2	2	Yes
M45	Co-feeding	M20	2	50.0%	1	2	1	Yes
M46	Co-feeding	M20	2	50.0%	1	1	0	No
		Total	90		27	80	17	13/26

Mouse ID[a]	Treatment[b]	Flat nymphs for infestation Origin[c]	Total1[d]	Prev[e]	Infect1[f]	Engorged nymphs recovered Total2[g]	Infect2[h]	Challenge[i]
M47	Systemic	M11	1	100.0%	1	1	1	Yes
M48	Systemic	M11	1	100.0%	1	0		No
M49	Systemic	M15	2	50.0%	1	2	0	No
M50	Systemic	M15	2	50.0%	1	1	0	No
M51	Systemic	M16	1	100.0%	1	1	1	Yes
M52	Systemic	M16	1	100.0%	1	1	0	No
M53	Systemic	M18	1	100.0%	1	1	1	Yes
M54	Systemic	M18	1	100.0%	1	1	1	Yes
M55	Systemic	M19	1	100.0%	1	1	0	No
M56	Systemic	M19	1	100.0%	1	1	1	Yes
M57	Systemic	M20	1	100.0%	1	1	1	Yes
M58	Systemic	M20	1	100.0%	1	1	1	Yes
		Total	14		12	12	7	7/12

Mouse ID[a]	Treatment[b]	Flat nymphs for infestation Origin[c]	Total1[d]	Prev[e]	Infect1[f]	Engorged nymphs recovered Total2[g]	Infect2[h]	Challenge[i]
M59	Control	M8	5	0.0%	0	5	0	No
M60	Control	M10	5	0.0%	0	5	0	No
M61	Control	M3	5	0.0%	0	3	0	No
		Total	15		0	13	0	0/3

[a] Mouse ID is the unique mouse identification number.

[b] Treatment is whether the mice were infested with co-feeding nymphs, systemic nymphs, or uninfected nymphs.

[c] Origin refers to the parental mouse (see Table S1) that generated the co-feeding or the systemic nymphs.

[d] Total1 is the number of nymphs that were placed on each mouse.

[e] Prevalence (Prev) is the expected proportion of Total1 ticks that are infected.

[f] Infect1 is the expected number of infected nymphs placed on the mouse (Total1*Prev).

[g] Total2 is the number of engorged nymphs recovered from each mouse.

[h] Infect2 is the number of Total2 ticks that tested positive for *B. afzelii*.

[i] Challenge is whether at least one engorged *B. afzelii*-infected nymph was recovered for each mouse (Yes) or not (No).

Infection status of mice infested with co-feeding and systemic nymphs: At 39 days PI, a blood sample and an ear tissue biopsy was taken from all the mice. At 42 days PI, all mice were infested with pathogen-free, xenodiagnostic *I. ricinus* larvae to measure mouse-to-tick (systemic) transmission. At 59 days PI, all mice were sacrificed and dissected for the heart, bladder, and skin of the stomach. The infection status of each mouse was determined using five criteria: presence of spirochetes in the ear, heart, bladder, ventral skin, and presence of *Borrelia*-specific IgG antibodies in the blood (Table S3). Only those mice testing positive for one or more of these five criteria were considered as infected with *B. afzelii* (Table S3). For the first four criteria, the presence of spirochetes in the ear, heart, bladder, and ventral skin, the threshold of classifying a mouse as being infected or clear depended on whether the spirochete load was greater than zero or equal to zero. For the fifth criterion, the presence of *Borrelia*-specific IgG antibodies, there was a clear separation in the range of the absorbance values for the uninfected mice (198–403 absorbance units) and the infected mice (2664–4471 absorbance units). In Table S3, all mice that were infected with *B. afzelii* tested positive for 3 or more of the 5 infection criteria. Mice that had not become infected following the nymphal infestation tested negative for all of the 5 infection criteria.

Production of low numbers of *B. afzelii*-positive xenodiagnostic nymphs by uninfected mice: Of the 14 mice that were not infected with *B. afzelii*, 13 mice produced 130 xenodiagnostic nymphs; mouse M46 died before the infestation with the xenodiagnostic larval ticks (Table S3). According to the qPCR, 92.3% (120/130) of these xenodiagnostic nymphs tested negative for *B. afzelii* and 7.7% (10/130) tested positive. To determine whether these nymphs contained *Borrelia* DNA, we amplified the *Borrelia ospC* gene and sequenced the amplicon. We succeeded in amplifying the *ospC* gene for 6 of these 10 nymphs and all six *ospC* gene sequences clustered with major *ospC* group A10. The 6 infected nymphs were produced by the following 5 uninfected mice: M27, M37, M40, M48 (2x), and M49 (Table S3). Of these 6 *B. afzelii*-positive ticks, 3 had high Cq values (≥38.5 cycles) and low spirochete loads (≤ 6.4 spirochetes per nymph). The remaining 3 ticks had lower Cq values (37.41, 35.02, 32.70) and higher spirochete loads (12.1, 67.2, 315.5

spirochetes per nymph). One possible explanation is that these 6 *B. afzelii*-infected ticks were somehow contaminated during the DNA extraction process. Another explanation is that these mice were challenged with infected nymphs, but that *B. afzelii* never managed to disseminate from the site of the tick bite and establish a systemic infection. At 42 days after the nymphal challenge, larval ticks feeding near the nymphal tick bite site acquired either live spirochetes and/or dead spirochete DNA. Regardless of their true infection status, these nymphs did not influence the results because the infection status of each mouse in Table S3 was based on five other infection criteria.

Table S3. The *B. afzelii* infection status is shown for the mice that were challenged with co-feeding nymphs, systemic nymphs, or uninfected control nymphs. The infection status is based on five criteria: *Borrelia*-specific IgG antibodies (ELISA) and presence of spirochetes in the following four tissues: ear, heart, bladder, and ventral skin. Also shown are the results from a xenodiagnostic assay that quantified systemic (host-to-tick) transmission of *B. afzelii*. All *B. afzelii*-infected mice tested positive for at least 3 of the 5 criteria whereas the uninfected mice tested negative for all five criteria.

Mouse ID[a]	Treatment[b]	ELISA[c]	Ear[d]	Heart[e]	Bladder[f]	Skin[g]	Xeno[h]	Criteria[i]	Status[j]
M21	Co-feeding	2,826	36	0	95	229	8/10 (80.0%)	4	Infected
M22	Co-feeding	2,689	70	168	347	1003	7/9 (77.8%)	5	Infected
M23	Co-feeding	2,671	69	182	431	1976	8/10 (80.0%)	5	Infected
M24	Co-feeding	209	0	0	0	0	1*/10 (10.0%)	0	Clear
M25	Co-feeding	392	0	NA	0	0	0/10 (0.0%)	0	Clear
M26	Co-feeding	198	0	0	0	0	0/10 (0.0%)	0	Clear
M27	Co-feeding	230	0	0	0	0	1/10 (10.0%)	0	Clear
M28	Co-feeding	238	0	0	0	0	1*/10 (10.0%)	0	Clear
M29	Co-feeding	2,765	41	0	236	382	8/10 (80.0%)	4	Infected
M30	Co-feeding	202	0	0	0	0	0/10 (0.0%)	0	Clear
M31	Co-feeding	3,344	65	38	722	1013	10/10 (100.0%)	5	Infected
M32	Co-feeding	2,664	59	216	138	964	9/10 (90.0%)	5	Infected
M33	Co-feeding	3,391	95	0	100	1046	5/10 (50.0%)	4	Infected
M34	Co-feeding	3,629	5	104	583	168	7/10 (70.0%)	4	Infected
M35	Co-feeding	4,043	16	0	190	394	7/10 (70.0%)	4	Infected
M36	Co-feeding	3,956	62	355	0	207	6/10 (60.0%)	4	Infected
M37	Co-feeding	403	0	0	0	0	1/10 (10.0%)	0	Clear

M38	Co-feeding	2,765	115	0	1046	0	7/10 (70.0%)	3	Infected
M39	Co-feeding	3,532	50	98	0	416	8/10 (80.0%)	4	Infected
M40	Co-feeding	234	0	0	0	0	2*/10 (20.0%)	0	Clear
M41	Co-feeding	3,578	123	0	455	146	9/10 (90.0%)	4	Infected
M42	Co-feeding	3,434	122	0	295	272	9/10 (90.0%)	4	Infected
M43	Co-feeding	3,683	92	149	1864	169	7/10 (70.0%)	5	Infected
M44	Co-feeding	4,471	24	0	166	1045	7/10 (70.0%)	4	Infected
M45	Co-feeding	3,074	36	0	104	658	9/10 (90.0%)	4	Infected
M46	Co-feeding	205	0	NA	0	0	Dead	0	Clear
M47	Systemic	3,780	28	67	713	133	6/10 (60.0%)	5	Infected
M48	Systemic	281	0	0	0	0	2/10 (20.0%)	0	Clear
M49	Systemic	234	0	0	0	0	1/10 (10.0%)	0	Clear
M50	Systemic	3,650	31	140	247	147	6/10 (60.0%)	5	Infected
M51	Systemic	3,964	31	0	406	710	7/10 (70.0%)	4	Infected
M52	Systemic	3,823	37	0	970	643	9/10 (90.0%)	4	Infected
M53	Systemic	3,096	43	0	306	0	5/10 (50.0%)	3	Infected
M54	Systemic	2,768	18	158	140	1130	7/10 (70.0%)	5	Infected
M55	Systemic	3,344	106	0	620	1289	Dead	5	Infected
M56	Systemic	3,481	23	0	0	336	10/10 (100.0%)	3	Infected
M57	Systemic	3,492	21	0	394	3035	6/10 (60.0%)	4	Infected
M58	Systemic	2,869	21	0	647	1115	9/10 (90.0%)	4	Infected
M59	Control	198	0	0	0	0	0/10 (0.0%)	0	Clear
M60	Control	306	0	0	0	0	0/10 (0.0%)	0	Clear
M61	Control	216	0	0	0	0	1*/10 (10.0%)	0	Clear

[a] Mouse ID is the unique mouse identification number.

[b] Treatment is whether the mice were infested with co-feeding nymphs, systemic nymphs, or uninfected nymphs.

[c] ELISA indicates the strength of the *B. afzelii*-specific IgG antibody response. The antibody response was measured as the integral of the absorbance at 652 nm over a period of 60 minutes.

[d] Ear is the number of spirochetes in the ear tissue biopsy (2 mm diameter).

[e] Heart is the number of spirochetes in 1 mg of heart tissue.

[f] Bladder is the number of spirochetes in 1 mg of bladder tissue.

[g] Skin is the number of spirochetes in 1 mg of skin tissue.

[h] Xeno is the number of infected nymphs divided by the total number of nymphs tested. These nymphs were fed as xenodiagnostic larval ticks on the mice on day 42 PI. The engorged xenodiagnostic larval ticks were allowed to moult into nymphs and tested for *B. afzelii* infection. There are 4 infected ticks marked with an asterisk (*) that tested positive for *B. afzelii* on the *flagellin* qPCR but that tested negative for the *ospC* PCR.

[i] Criteria is the number of phenotypes for which the mouse tested positive for *B. afzelii* infection.

[j] Status refers to whether a mouse was considered to be infected with *B. afzelii* or not.

Comparison of infection phenotype between infected mice and uninfected mice: To show that the uninfected and infected mice had different infection phenotypes, independent samples t-tests were used to compare the following three response variables: (1) *Borrelia*-specific IgG antibody response, (2) spirochete load in the ear tissues, and (3) proportion of infected xenodiagnostic nymphs. The mean *Borrelia*-specific IgG antibody response of the infected mice (3327; range = 2664–4471 absorbance units) was 13.3 times higher than that of the uninfected mice (246; range = 198–403 absorbance units) and this difference was highly significant (Figure S1; t-test: $t = 43.045$, $df = 39$, $p < 0.001$). The mean spirochete load in the ear tissues of the infected mice (41; range = 5–147 spirochetes per ear tissue biopsy) was much higher than that of uninfected mice (0; range = 0–0 spirochetes per ear tissue biopsy) and this difference was highly significant (Figure S2; t-test: $t = 18.267$, $df = 39$, p-value < 0.001). The mean proportion of infected xenodiagnostic ticks was much higher in the infected mice (0.757, range = 0.50–1.00 infected nymphs) than the uninfected mice (0.077, range = 0.00–0.200 infected nymphs) and this difference was highly significant (Figure S3; t-test: $t = 16.196$, $df = 37$, $p < 0.001$). These comparisons show that infected and uninfected mice have very different infection phenotypes with respect to these three criteria. The comparison of the other 3 infection criteria, spirochete load in the heart, bladder, and ventral skin, between uninfected and infected mice gave similar results (data not shown).

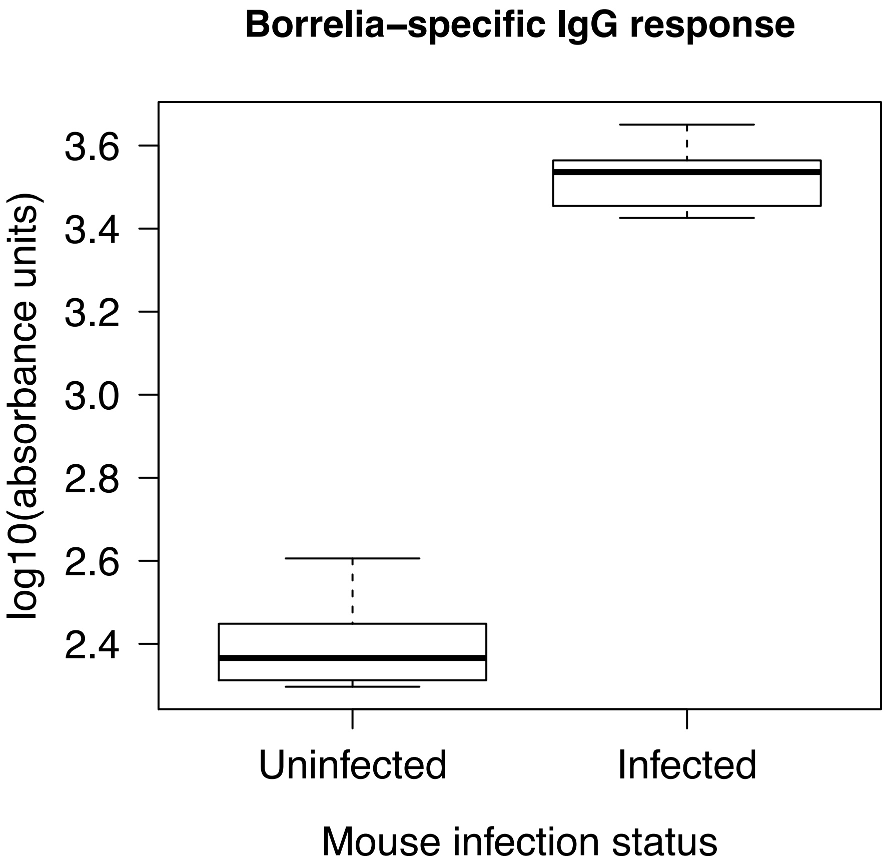

Figure S1. The infected mice (n = 27) have a much stronger IgG antibody response against *Borrelia afzelii* than the uninfected mice (n = 14). The uninfected mice include 3 control mice that were infested with uninfected *I. ricinus* nymphs. The strength of the *Borrelia*-specific IgG antibody response was measured using a commercial ELISA kit. Shown are the medians (black line), the 25th and 75th percentiles (edges of the box), the minimum and maximum values (whiskers), and the outliers (circles).

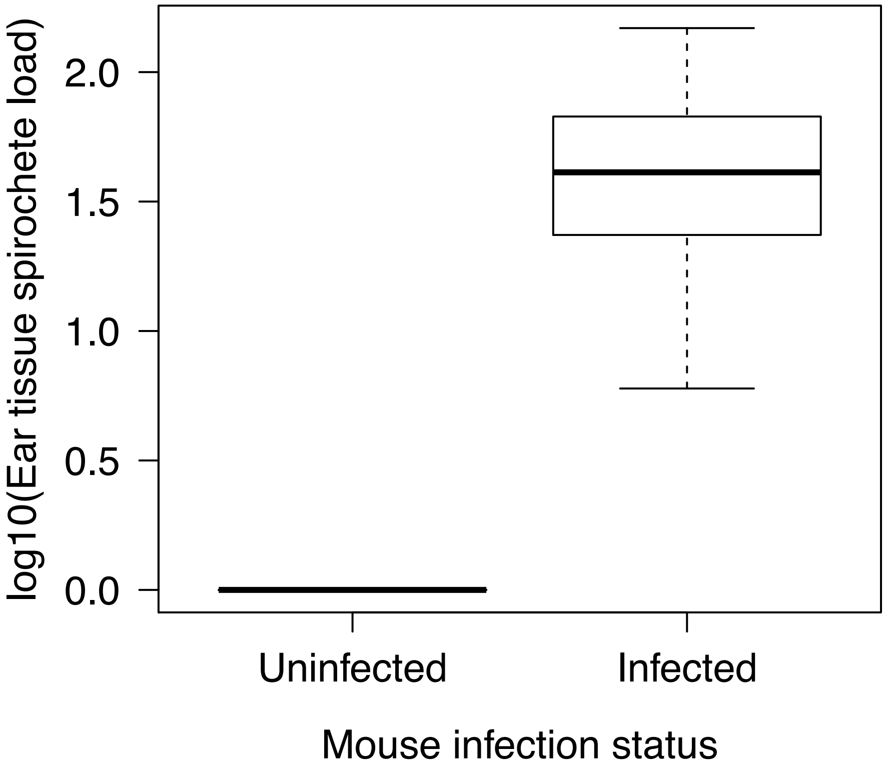

Figure S2. The spirochete load of the ear tissue ranged between 5–147 spirochetes per 2 mm biopsy for the infected mice (n = 27), whereas it was 0 for all the uninfected mice (n = 14). The uninfected mice included 3 control mice that were infested with uninfected *I. ricinus* nymphs. The presence of *B. afzelii* in the ear tissue suggests that the spirochetes migrated from the skin on the back (where the nymphal challenge took place) to the head and therefore that they established a systemic infection in the mouse. Shown are the medians (black line), the 25th and 75th percentiles (edges of the box), the minimum and maximum values (whiskers), and the outliers (circles).

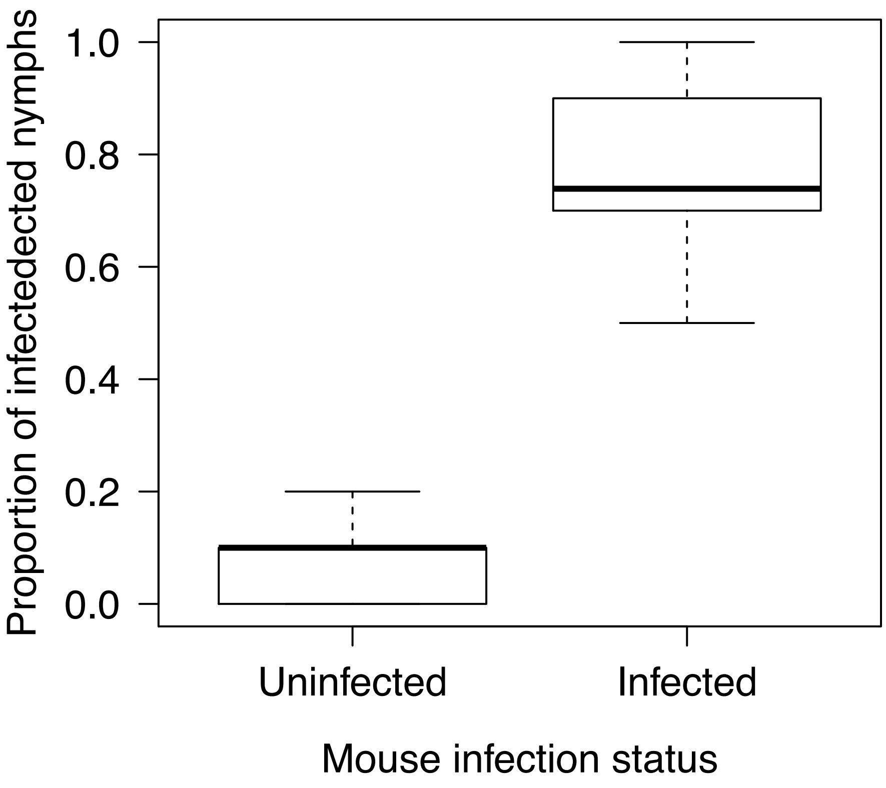

Figure S3. The infected mice (n = 27) produced a much higher proportion of *B. afzelii*-infected xenodiagnostic ticks than the uninfected mice (n = 14). The uninfected mice include 3 control mice that were infested with uninfected *I. ricinus* nymphs. Eight of the 14 uninfected mice produced a total of 10 nymphs that tested positive on the *flagellin* qPCR. These uninfected mice are responsible for the low percentages of infected ticks (10.0–20.0%). Of these 10 nymphs, 4 individuals tested negative for the *ospC* PCR, whereas the other six individuals contained *ospC* major group A10. Shown are the medians (black line), the 25th and 75th percentiles (edges of the box), the minimum and maximum values (whiskers), and the outliers (circles).

Total spirochete load in mice infected with *B. afzelii*: For the subset of mice that were infected with *B. afzelii*, the total spirochete load was calculated for the heart, bladder, and skin. The heart and bladder of each mouse had been weighed to the nearest mg. The mass of the skin was calculated as 16% of the mouse body mass. The total spirochete load for each of the three organs was calculated by multiplying the spirochete density per milligram of tissue by the mass of the tissue. The spirochete loads were then summed for the three organs (heart, bladder, and skin) to estimate the number of spirochetes present in the entire mouse. The mean spirochete load was 954,468 spirochetes per mouse (95% CI: 812,310 to 1,121,506 spirochetes per mouse). A recent study estimated that *I. ricinus* nymphs inoculate ~100 spirochetes into the vertebrate host during the nymphal blood meal [1]. These estimates suggest that the *B. afzelii* population increased 10,000-fold in the mice after inoculation by the nymphal tick.

References

1 Kern, A. *et al*. Tick saliva represses innate immunity and cutaneous inflammation in a murine model of Lyme disease. *Vector-Borne Zoonot*. **11**, 1343-1350 (2011).
